# Plasmonic-Induced Transparencies in an Integrated Metaphotonic System

**DOI:** 10.3390/nano12101701

**Published:** 2022-05-16

**Authors:** Fernando López-Rayón, Maximino L. Arroyo Carrasco, René I. Rodríguez-Beltrán, Rafael Salas-Montiel, Ricardo Téllez-Limón

**Affiliations:** 1 Facultad de Ciencias Físico-Matemáticas, Benemérita Universidad Autónoma de Puebla, Av. San Claudio y 18 Sur, San Manuel, Puebla 72570, Mexico; fernando.lopezrayon@alumno.buap.mx (F.L.-R.); marroyo@fcfm.buap.mx (M.L.A.C.); 2 CONACYT-Centro de Investigación Científica y de Educación Superior de Ensenada, Unidad Monterrey, Alianza Centro 504, PIIT, Apodaca 66629, Mexico; rrodrigu@cicese.mx; 3 Laboratory Light, Nanomaterials and Nanotechnologies, L2n CNRS EMR 7004, Université de Technologie de Troyes, 10004 Troyes, France; rafael.salas_montiel@utt.fr

**Keywords:** integrated optics, nanophotonics, localized surface plasmons, coupled nanoparticles, plasmon induced transparency, metaphotonics

## Abstract

In this contribution, we numerically demonstrate the generation of plasmonic transparency windows in the transmission spectrum of an integrated metaphotonic device. The hybrid photonic–plasmonic structure consists of two rectangular-shaped gold nanoparticles fully embedded in the core of a multimode dielectric optical waveguide, with their major axis aligned to the electric field lines of transverse electric guided modes. We show that these transparencies arise from different phenomena depending on the symmetry of the guided modes. For the TE${}_{0}$ mode, the quadrupolar and dipolar plasmonic resonances of the nanoparticles are weakly coupled, and the transparency window is due to the plasmonic analogue of electromagnetically induced transparency. For the TE${}_{1}$ mode, the quadrupolar and dipolar resonances of the nanoparticles are strongly coupled, and the transparency is originated from the classical analogue of the Autler–Townes effect. This analysis contributes to the understanding of plasmonic transparency windows, opening new perspectives in the design of on-chip devices for optical communications, sensing, and signal filtering applications.

## 1. Introduction

Integrated hybrid photonic–plasmonic devices are systems that combine dielectric photonic waveguides with plasmonic nanostructures, reducing intrinsic optical losses when light propagates through metals [1,2,3,4,5,6]. These hybrid systems have been employed for different on-chip applications, such as subwavelength-guided modes with continuous and periodic plasmonic nanostructures [7,8,9,10,11,12,13], optical switching devices [14,15,16], nanoscale light sources [17,18,19], structured light generation [20], second harmonic generation [21], and wavelength and mode converters [22,23,24], to mention a few.

For some applications, such as optical communications and signal filtering, it is desired to reduce the inherent broad spectral bandwidth of plasmonic resonances. A possible way to produce sharp dips in the transmission lines of the integrated systems is by coupling nano resonators with the waveguides, being obtained transparency windows around the natural frequencies of the resonators [25,26,27]. These transparency windows can be understood through a classical two-coupled harmonic oscillators model given by the relationship

(1) $$i\omega \left(\begin{array}{c} x_{1}\\ x_{2} \end{array}\right)=\left(\begin{array}{cc} i\omega_{1}+\gamma_{1} & i\kappa \\ i\kappa & i\omega_{2}+\gamma_{2} \end{array}\right)\left(\begin{array}{c} x_{1}\\ x_{2} \end{array}\right),$$

where $x_{j}\left(t\right)=x_{j}e^{i\omega t}$ (for j=1,2) represent the amplitude of the oscillators, $\omega_{j}$ the natural frequencies, $\gamma_{j}$ the damping coefficients, and κ the coupling constant that rules the coupling regime of the system [25,27,28,29,30]. When the damping is lower than the oscillators energy exchange, i.e., the coupling strength is larger than the energy losses ($\kappa >>\gamma_{1},\gamma_{2}$), the system is strongly coupled, leading to the so-called Autler–Townes effect, producing hybrid resonances with symmetric and antisymmetric eigenfrequencies [31]. If the coupling strength is lower than the damping ($\kappa <<\gamma_{1},\gamma_{2}$), the system is weakly coupled, and no mode hybridization is obtained. Instead, by breaking the symmetry of the oscillators, interference of dark and bright states would lead to the classical analogue of electromagnetically induced transparency (EIT), generating, once more, sharp resonances in the transmission spectra [26,28].

For integrated metaphotonic systems, plasmonic transparency windows in the transmission spectra of the systems, around the plasmonic resonance of the cavities, have been demonstrated either by mode hybridization or through the plasmonic analogue of EIT [32,33,34]. A large majority of these works use non-symmetric nanocavities integrated to plasmonic waveguides [32,35,36,37,38], or by exciting localized surface plasmons (LSP) of non-symmetric metallic nanoparticles excited with the evanescent field of guided modes [39,40].

Another possibility to induce plasmonic transparencies is by fully embedding nanoparticles (NPs) in the core of the waveguide and taking advantage of the symmetry of the guided modes to excite LSP resonances. These confined architectures reduce scattering losses and increase the contrast of dips in the transmission lines [41,42,43,44]. However, they have not been exploited for the generation of plasmonic transparency windows.

**Figure 1:**
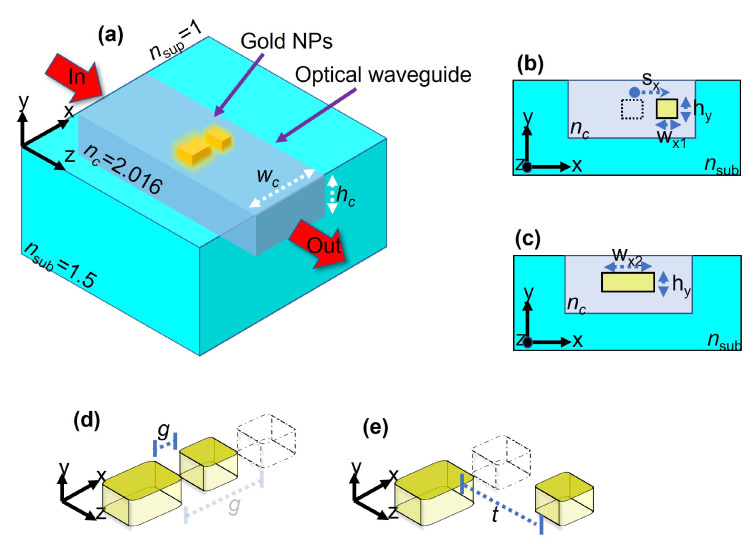
(**a**) Schematic of two gold NPs embedded in the core of a Si${}_{3}$N${}_{4}$ waveguide. (**b**) Short NP laterally displaced by a distance $s_{x}$ from the center of the core to the center of the NP. (**c**) Long NP integrated at the center of the waveguide. (**d**) Two NPs laterally separated by a gap *g* along the *x* axis. (**e**) Two NPs longitudinally separated by a distance *t* along the *z* axis.

In this contribution, we numerically demonstrate that a system consisting of two NPs embedded in the core of a silicon nitride (Si${}_{3}$N${}_{4}$) multimode dielectric waveguide, as depicted in Figure 1a, can be used to generate transparency windows around the plasmonic resonance of the NPs. Furthermore, we demonstrate that due to the symmetry of the guided modes and the nanoparticles, transparency windows can be obtained for both TE${}_{0}$ and TE${}_{1}$ modes of the waveguide, but they arise from different phenomena: for the TE${}_{0}$ mode, the transparency is originated from the excitation of the plasmonic analogue of EIT, while for the TE${}_{1}$ mode, the transparency results from the strong coupling between the plasmonic resonances, analogue to the Autler–Townes effect.

## 2. Description and Principle of Operation of the System

**Figure 2:**
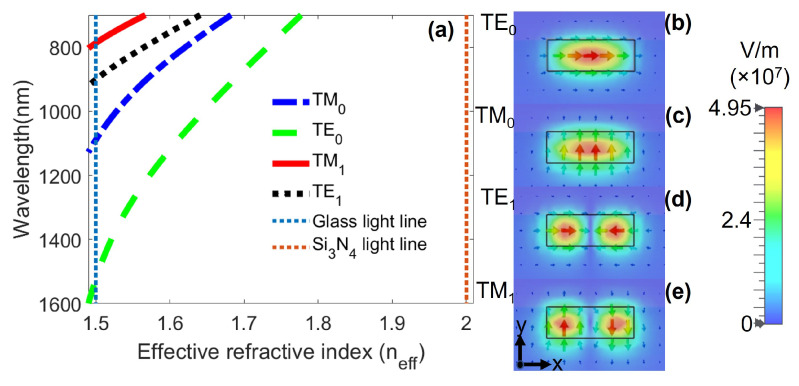
(**a**) Dispersion curves of the $Si_{3}N_{4}$ waveguide. The cutoff wavelengths for the fundamental TE${}_{0}$ and TM${}_{0}$ modes are $\lambda_{TE_{0c}}=1523$ nm and $\lambda_{TM_{0c}}=1089$ nm, respectively. The TE${}_{1}$ mode has a cutoff wavelength $\lambda_{TE_{1c}}=901$ nm and for the TM1 mode $\lambda_{TM_{1c}}=787$ nm. (**b**–**e**) Intensity maps and electric field distribution for the modes at $n_{eff}=1.5$.

The proposed system is an asymmetric dimer constituted by two rectangular-shaped gold NPs fully embedded in the core of a multimode waveguiding platform (Figure 1a–c). The waveguide consists of a dielectric rectangular core of refractive index $n_{c}=2.016$ (Si${}_{3}$N${}_{4}$), width $w_{c}=750$ nm along the *x* axis, and height $h_{c}=250$ nm along the *y* direction. The core is buried in a glass substrate of refractive index $n_{sub}=1.5$ and guided modes propagate in the *z* direction (Figure 1a). The superstrate was considered as air ($n_{sup}=1.0$). In the spectral range from 700 nm to 1523 nm, this waveguide supports the propagation of four modes, as can be seen in the dispersion curves of Figure 2a. The cut-off wavelengths for the TE${}_{0}$ (green dashed), TM${}_{0}$ (blue dotted-dashed), TE${}_{1}$ (black dotted), and TM${}_{1}$ (red continuous) modes are $\lambda_{TE_{0}c}=1523$ nm, $\lambda_{TM_{0}c}=1089$ nm, $\lambda_{TE_{1}c}=901$ nm, and $\lambda_{TM_{1}c}=787$ nm, respectively. The vertical lines correspond to light-lines of the Si${}_{3}$N${}_{4}$ core (red dotted) and glass substrate (blue dotted). Figure 2b–e show the intensity maps and electric field lines for each mode at $n_{eff}=1.5$. As observed, the electric field for the TE${}_{0}$ and TE${}_{1}$ modes is mainly oriented along the horizontal *x* axis (Figure 2b,d), but TE${}_{1}$ presents mirror symmetry respect to the *y* axis. For TM${}_{0}$ and TM${}_{1}$ modes, the electric field is vertically oriented (Figure 2c,e).

The asymmetric dimer formed by the two rectangular-shaped gold NPs was placed as follows: one NP of width $w_{x1}=60$ nm (short NP) is placed at a distance $s_{x}$ measured from the center of the NP to the center of the waveguide, while the other one of width $w_{x2}=150$ nm (long NP) is located at the center of the waveguide. Both NPs have the same height $h_{y}=30$ nm and length $l_{z}=50$ nm, the corners of the NPs were rounded with a radius of r = 5 nm, and the dielectric function of gold was computed from the Drude–Lorentz model as described in reference [45]. The numerical analysis of the system was performed using the finite integration technique [46] with the commercial software CST Studio Suite 2020 (Dassault Systems, Vélizy-Villacoublay, France). We defined an excitation port at the input of the waveguide in a computational window of 3μm ×2.4μm to compute the guided modes and dispersion curves. The guided modes were launched throughout the waveguide of length 4μm and the transmitted signal was measured with a port monitor at the end of the waveguide. The computational window was surrounded with perfectly matched layers to prevent spurious reflections.

The operation principle of the system is as follows: the photonic modes supported by the waveguide are launched at the input. When propagating, symmetry-compatible modes will excite LSP resonances of the NPs. The transmission spectrum measured at the output of the waveguide, presents depths associated with the excitation of the plasmonic resonances. The size of each NP was tuned such that, when individually excited, both long and short NPs presented LSP resonances at similar wavelengths in the transmission spectrum. When the NPs are properly placed, their resonances are coupled, creating transparency windows in the transmission spectrum around the resonance wavelength of the LSP. However, as will be demonstrated, due to the symmetry of the photonic modes, these transparencies arise from different physical phenomena.

## 3. Results

### 3.1. Single Nanoparticle

#### 3.1.1. Short Nanoparticle

**Figure 3:**
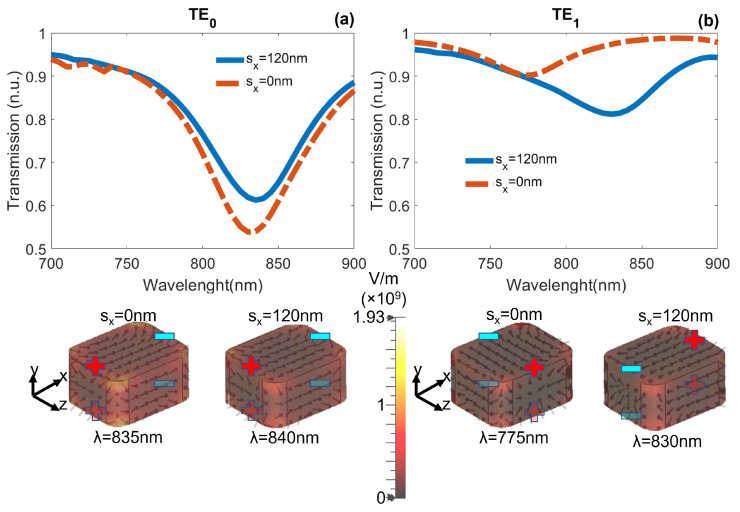
Transmission spectra for (**a**) TE${}_{0}$ and (**b**) TE${}_{1}$ modes with a short NP embedded at the center ($s_{x}=0$ nm) and out of the center ($s_{x}=120$ nm) of the core. The lower schematics show the electric field lines on the NP: dipoles along *x* for the TE${}_{0}$ mode, and dipoles along *z* and *x* for the TE${}_{1}$ mode.

We measured the transmission spectra for the modes propagating through the waveguide when the short NP ($w_{x1}=60$ nm) was placed in the core and laterally shifted along the *x* axis by a distance $s_{x}=120$ nm, measured from the center of the core to the center of the NP (Figure 1b). As demonstrated in reference [41], due to the mode symmetry and geometry of the NP, only TE${}_{0}$ and TE${}_{1}$ modes excite plasmonic resonances for this NP in the spectral range of interest. The transmission spectra for TE${}_{0}$ and TE${}_{1}$ modes are shown in Figure 3a,b, respectively. The red dashed curve corresponds to the transmission spectrum when the NP is placed at the center ($s_{x}=0$ nm), while the blue continuous curve is when it is shifted from the center ($s_{x}=120$ nm). The lower schematics show the distribution of the electric field lines on the NP at dips of both transmission spectra.

As the electric field of the TE${}_{0}$ mode is mainly horizontally oriented, the NP presents a dipolar LSP resonance oriented along the *x* axis. When the NP is at the center ($s_{x}=0$ nm), this resonance reduces the transmission signal to 53% for a wavelength λ=835 nm. When the NP is out of the center ($s_{x}=120$ nm), the minimum in transmission is slightly shifted to λ=840 nm and the signal drops to 61% (Figure 3a).

Because of the mirror symmetry of TE${}_{1}$ mode, when the NP is at the center ($s_{x}=0$ nm), there is no excitation of the dipolar LSP resonance along *x*, but along the *z* direction. This resonance leads to a small reduction in the transmission spectrum (90% of light is transmitted) at a wavelength λ=775 nm. However, when the NP is laterally shifted ($s_{x}=120$ nm), the portion of the electric field surrounding the NP is symmetric, exciting a dipolar LSP resonance along *x*. This resonance reduces the transmission signal to 81% around λ=830 nm (Figure 3b).

#### 3.1.2. Long Nanoparticle

**Figure 4:**
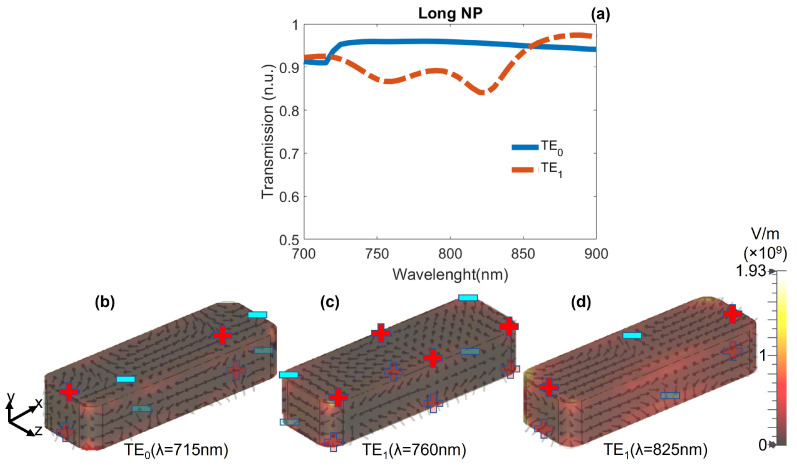
(**a**) Transmission spectra for the TE${}_{0}$ and TE${}_{1}$ modes exciting a long NP ($w_{x2}=150$ nm) placed at the center of the core. (**b**–**d**) Schematics of the electric field lines on the NP at the minimum values for the TE${}_{0}$ and TE${}_{1}$ spectra.

In Figure 1c is depicted the system consisting of a longer NP of width $w_{x2}=150$ nm, placed at the center of the core of the waveguide. The height and length remain the same. In Figure 4 are plotted the transmission spectra for the TE${}_{0}$ (blue continuous) and TE${}_{1}$ (red dashed) modes. For the TE${}_{0}$ guided mode, the transmitted signal is almost flat, with a small depth around λ=715 nm, corresponding to the excitation of a hexapolar LSP. For the TE${}_{1}$ mode, the transmission spectrum exhibits two depths, corresponding to a higher-order and a quadrupolar LSP resonance located at λ=760 nm (transmission of 87%) and at λ=825 nm (transmission of 84%), respectively. This situation means that the quadrupolar LSP resonance of the long NP is only accessible if the guided mode is asymmetric respect to the long axis of the NP. Hence, the quadrupolar LSP is similar to a dark state for the TE${}_{0}$ mode.

### 3.2. Two Nanoparticles

When integrating small and long NPs in the core of the waveguide, it is expected that their LSP resonances will couple, as both NPs present minimum transmission values for TE${}_{0}$ and TE${}_{1}$ modes around the same wavelength (λ=830 nm). As the coupling is ruled by the separation distance between the NPs, we computed the transmission spectra for TE${}_{0}$ and TE${}_{1}$ modes when the small NP is laterally (along the *x* axis as in Figure 1d) and longitudinally (along the *z* direction as in Figure 1e) shifted from the center of the core. The long NP rests at the center of the core; the following results were obtained.

#### 3.2.1. Lateral Shift

We placed the long NP at the center of the core, and the short NP was placed at distances of $s_{x}=$ 120, 155, 205 nm along the *x* axis, forming gaps between the NPs of g= 15, 50, 100 nm, respectively, as illustrated in Figure 1d.

**Figure 5:**
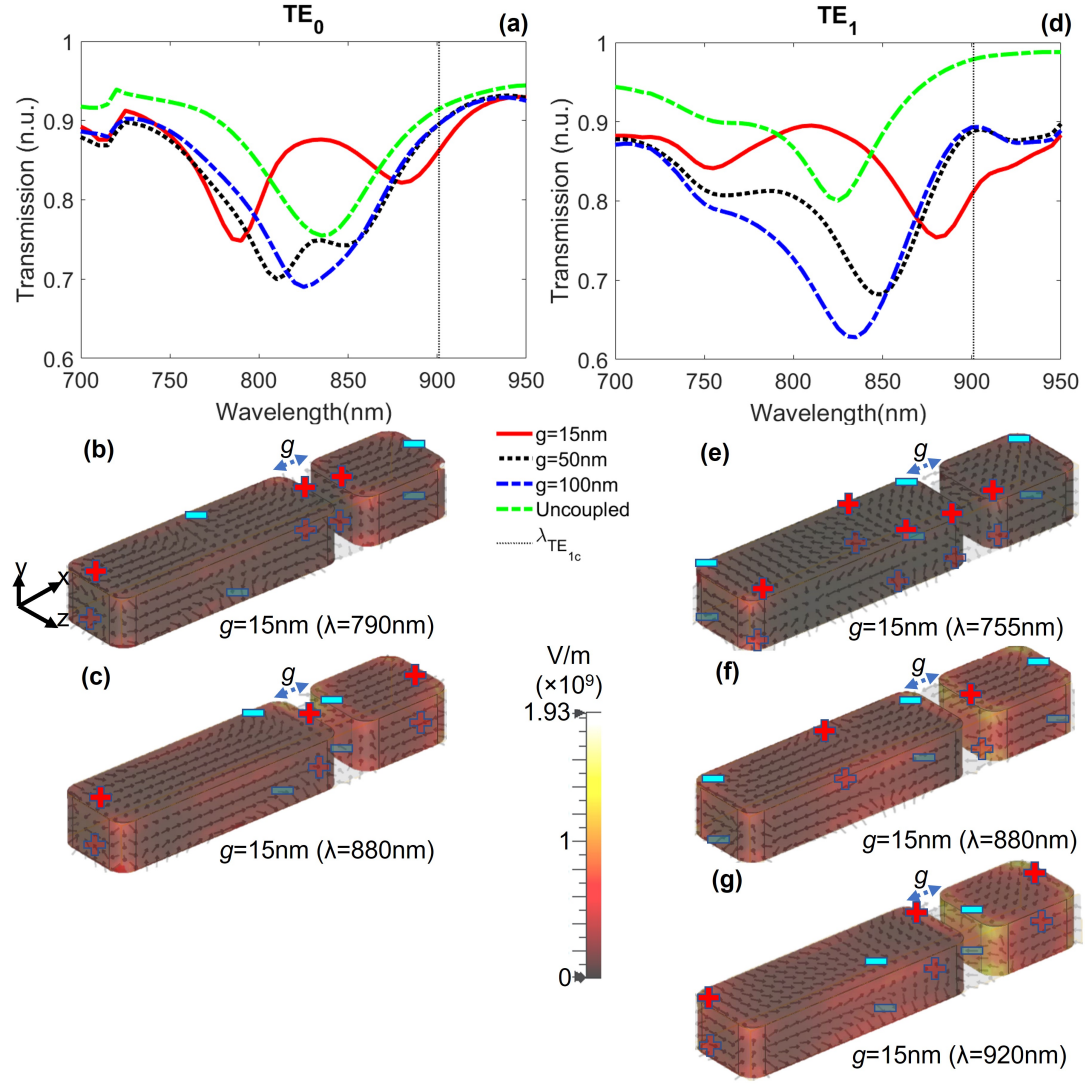
Transmission spectra with a dimer of gaps g=15,50,100 nm. (**a**) For the TE${}_{0}$ mode, the EIT-like effect is observed around λ=835 nm (red continuous curve). As the gap *g* increases, the transparency window tends to disappear. (**b**,**c**) Electric field lines on the NPs for TE${}_{0}$ mode. (**d**) For the TE${}_{1}$ mode, the transparency effect is obtained around λ=810 nm (red continuous curve) due to strong coupling of the NPs. The wider the gap, the weaker the transparency effect. (**e**–**g**) Electric field lines on the NPs for TE${}_{1}$ mode.

Figure 5a shows the transmission spectrum for the TE${}_{0}$ mode. When g=15 nm, the transmission spectrum shows a local transparency window around a wavelength λ=835 nm, close to the LSP resonance (red continuous curve) with a maximum transmission of 87%; also, two depths are generated at λ=790 nm and λ=880 nm, separated by a spectral distance Δλ=90 nm. When the gap is increased at g=50 nm, these depths approach each other (Δλ=40 nm), reducing the contrast in the transparency window (black dotted curve). When the gap is increased to g=100 nm, the NPs are not coupled and no transparency window is observed (blue dashed curve). Instead, the transmission spectrum is similar to the addition of the transmission spectra of the NPs individually excited (green dotted-dashed curve), i.e., the sum of blue continuous curves in Figure 3a and Figure 4a.

This transparency window is due to the plasmonic analogue of EIT. This can be clarified by observing the distribution of the charges in the NPs of Figure 5b,c at λ=790 nm and λ=880 nm (the two depths of red continuous curve in Figure 5a). At these wavelengths, dipolar and quadrupolar LSP for the short and long NPs are excited, respectively. This means that the quadrupolar resonance becomes accessible for the TE${}_{0}$ mode. In other words, the transparency window is the result of the interference between dark (quadrupolar resonance) and bright (dipolar resonance) states [47].

In Figure 5d are plotted the transmission spectra for the TE${}_{1}$ mode for g=15,50,100 nm (red continuous, black dotted, and blue dashed curves, respectively). A transparency window is also observed around λ=810 nm, with a transmission of 89%. For g=15 nm, three minimum values are observed. The first one, at λ=755 nm, corresponds to the excitation of a higher-order LSP resonance of the long NP and dipolar mode in the short one (Figure 5e). The minima at λ=880 nm and λ=920 nm correspond to out-of-phase antisymmetric (Figure 5f) and symmetric (Figure 5g) LSP resonances due to the strong coupling between quadrupolar and dipolar modes of the NPs, analogous to the Autler–Townes effect [28]. However, as the cut-off wavelength for the TE${}_{1}$ mode is around λ=901 nm, the symmetric mode cannot be accessed by the system. Hence, the transparency window observed at λ=810 nm is the result of the excitation of the higher-order and antisymmetric resonances of the NPs. When the gap is increased, the antisymmetric mode is blue-shifted and the transparency window tends to disappear, until the spectrum becomes similar to the addition of the spectra of the NPs individually illuminated (green dotted-dashed curve), i.e., as if the NPs were not coupled.

#### 3.2.2. Longitudinal Shift

**Figure 6:**
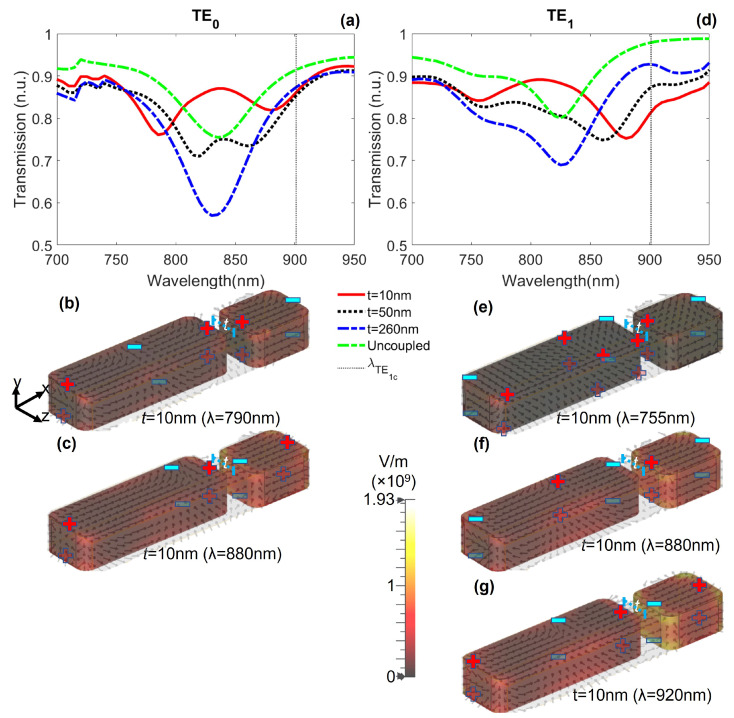
Transmission spectra for a dimer with the short NP longitudinally displaced by distances t=10,50,260 nm. (**a**) For the TE${}_{0}$ mode, the EIT-like effect is present around λ=835 nm. (**b**,**c**) Electric field lines on the NPs for TE${}_{0}$ mode. (**d**) For the TE${}_{1}$ mode, the transparency effect is observed around λ=810 nm due to strong coupling of the NPs. The larger the value of *t*, the weaker the transparency window, demonstrating that, in both cases, the transparency effect is possible only if the SVEA is satisfied. (**e**–**g**) Electric field lines on the NPs for TE${}_{1}$ mode.

To observe the influence of a longitudinal shift on the transparency window, we displaced the NPs at center-to-center distance of t=10,50,250 nm with respect to each other along the propagation *z* axis (Figure 1e). We kept the short NP at a constant lateral distance $s_{x}=120$ nm (g=15 nm). The transmission spectra for TE${}_{0}$ and TE${}_{1}$ modes are shown in Figure 6a,d, respectively. For both modes, as *t* increases, the interaction between the LSP resonances of the NPs is reduced and the transmission spectra shows no transparency window. For the TE${}_{0}$ mode, if the longitudinal separation between the NPs is shorter than the wavelength of the guided mode, the slowly-varying envelope approximation (SVEA) for dipolar transitions is satisfied, in agreement with the statements for the excitation of plasmonic analogue of EIT [48,49], leading to a transparency window around λ=835 nm with a maximum transmission of 87% (Figure 6a, red continuous curve). For the TE${}_{1}$ mode, the transparency window observed around λ=810 nm with a maximum transmission of 89% (Figure 6d, red continuous curve) is because of the excitation of the higher-order and antisymmetric plasmonic resonances. As the longitudinal shift is increased, the NPs are not strongly coupled and the transparency is lost, as observed in black dotted (t=50 nm) and blue dot-dashed (t=260 nm) curves in Figure 6d.

## 4. Conclusions

By integrating a metallic NP in the core of the dielectric waveguide, the transmission spectrum of guided modes presents depths around the excitation wavelength of LSP resonances. The LSP can be excited if the symmetry of guided modes is such that its electric field is parallel to the principal axis of the NP.

When individually integrated into the waveguide, the short NP exhibited a dipolar resonance when excited with both TE${}_{0}$ and TE${}_{1}$ modes, even when shifted from the center of the core of the waveguide. When placed at the center of the core of the waveguide, the long NP showed quadrupolar and higher-order resonances when illuminated with the TE${}_{1}$ mode, but no resonance was excited with the TE${}_{0}$ mode. Hence, the quadrupolar mode of the long NP is a dark state for the TE${}_{0}$ mode. The width of the NPs was tuned such that the wavelength of the dipolar resonance of the short NP laterally shifted was close to that of the quadrupolar resonance of the long NP at the center of the waveguide. The height and length were the same for both NPs and as these dimensions are too short, no LSP resonance was excited for the TM${}_{0}$ or TM${}_{1}$ modes [41].

When both NPs were integrated into the waveguide, a transparency window in the transmission spectra for TE${}_{0}$ and TE${}_{1}$ modes appeared around the resonance wavelength for the dipolar and quadrupolar resonances. This transparency occurred if the lateral separation between the NPs was short enough to allow the interaction between their LSP. When longitudinally shifted, the transparency occurred when their separation distance was shorter than half of the wavelength of the guided mode. However, the reason why the transparency originates is different for each guided mode.

For the TE${}_{0}$ mode, the transparency window is caused by the interference between the dipolar LSP of the short NP and the non-accessible quadrupolar mode of the long NP. That is to say, the interference between bright and dark states, namely, the plasmonic analogue of EIT. For the TE${}_{1}$ mode, both LSP resonances are accessible, hence the transparency arises from the strong coupling between dipolar and quadrupolar modes. For our proposed system, the transparency was formed by the higher-order LSP resonance of the long NP and the antisymmetric mode of coupled NPs (the symmetric mode was observed for a wavelength beyond the cut-off wavelength of the TE${}_{1}$ mode).

Without loss of generality, the architecture of the integrated metaphotonic system can be modified to tune the transparency wavelength and the spectral range of interest, as long as the aforementioned conditions are satisfied. The comprehensive analysis performed not only contributes to the understanding of the physics behind coupled LSP resonances in integrated systems, but also for the development of minimalistic integrated optical systems for on-chip applications, such as optical communications, signal filtering, and even biosensing.

## Data Availability

The data presented in this study are available on request from the corresponding author.

## References

[1] Benson O. (2011). Assembly of hybrid photonic architectures from nanophotonic constituents. Nature.

[2] Luo Y., Chamanzar M., Apuzzo A., Salas-Montiel R., Nguyen K.N., Blaize S., Adibi A. (2015). On-Chip Hybrid Photonic–Plasmonic Light Concentrator for Nanofocusing in an Integrated Silicon Photonics Platform. Nano Lett..

[3] Fang Y., Sun M. (2015). Nanoplasmonic waveguides: Towards applications in integrated nanophotonic circuits. Light. Sci. Appl..

[4] Dubrovina N., Fan Y., Roux X.L., de Lustrac A., Lupu A. Guided wave metamaterials for integrated optics applications. Proceedings of the 2017 19th International Conference on Transparent Optical Networks (ICTON).

[5] Zhang J., Zhang L., Xu W. (2012). Surface plasmon polaritons: Physics and applications. J. Phys. D Appl. Phys..

[6] Pluchery O., Vayron R., Van K.M. (2011). Laboratory experiments for exploring the surface plasmon resonance. Eur. J. Phys..

[7] Chamanzar M., Xia Z., Yegnanarayanan S., Adibi A. (2013). Hybrid integrated plasmonic-photonic waveguides for on-chip localized surface plasmon resonance (LSPR) sensing and spectroscopy. Opt. Express.

[8] Nezhad M.P., Tetz K., Fainman Y. (2004). Gain assisted propagation of surface plasmon polaritons on planar metallic waveguides. Opt. Express.

[9] Richards D., Zayats A., Krenn J.R., Weeber J.C. (2004). Surface plasmon polaritons in metal stripes and wires. Philos. Trans. R. Soc. Lond. Ser. A Math. Phys. Eng. Sci..

[10] Melikyan A., Kohl M., Sommer M., Koos C., Freude W., Leuthold J. (2014). Photonic-to-plasmonic mode converter. Opt. Lett..

[11] Février M., Gogol P., Aassime A., Mégy R., Delacour C., Chelnokov A., Apuzzo A., Blaize S., Lourtioz J.M., Dagens B. (2012). Giant coupling effect between metal nanoparticle chain and optical waveguide. Nano Lett..

[12] Téllez-Limón R., Salas-Montiel R. (2021). Nanowires Integrated to Optical Waveguides.

[13] Tellez-Limon R., Blaize S., Gardillou F., Coello V., Salas-Montiel R. (2020). Excitation of surface plasmon polaritons in a gold nanoslab on ion-exchanged waveguide technology. Appl. Opt..

[14] Tang B., Dai L., Jiang C. (2011). Electromagnetically induced transparency in hybrid plasmonic-dielectric system. Opt. Express.

[15] Foroutan S., Rostami G., Dolatyari M., Rostami A. (2017). All-Optical switching in metal nanoparticles plasmonic waveguide using EIT phenomenon. Optik.

[16] Hajshahvaladi L., Kaatuzian H., Danaie M. (2018). Design and analysis of a plasmonic demultiplexer based on band-stop filters using double-nanodisk-shaped resonators. Opt. Quantum Electron..

[17] Yang A., Wang D., Wang W., Odom T.W. (2017). Coherent light sources at the nanoscale. Annu. Rev. Phys. Chem..

[18] Bermúdez-Ureña E., Tutuncuoglu G., Cuerda J., Smith C.L.C., Bravo-Abad J., Bozhevolnyi S.I., Fontcuberta i Morral A., García-Vidal F.J., Quidant R. (2017). Plasmonic waveguide-integrated nanowire laser. Nano Lett..

[19] Koenderink A.F. (2017). Single-photon nanoantennas. ACS Photon..

[20] Xie Z., Lei T., Li F., Qiu H., Zhang Z., Wang H., Min C., Du L., Li Z., Yuan X. (2018). Ultra-broadband on-chip twisted light emitter for optical communications. Light. Sci. Appl..

[21] Ferrari L., Wu C., Lepage D., Zhang X., Liu Z. (2015). Hyperbolic metamaterials and their applications. Prog. Quantum Electron..

[22] Tuniz A., Bickerton O., Diaz F.J., Käsebier T., Kley E.B., Kroker S., Palomba S., de Sterke C.M. (2020). Modular nonlinear hybrid plasmonic circuit. Nat. Commun..

[23] Li Z., Kim M.H., Wang C., Han Z., Shrestha S., Overvig A.C., Lu M., Stein A., Agarwal A.M., Lončar M. (2017). Controlling propagation and coupling of waveguide modes using phase-gradient metasurfaces. Nat. Nanotechnol..

[24] Guo R., Decker M., Setzpfandt F., Gai X., Choi D.Y., Kiselev R., Chipouline A., Staude I., Pertsch T., Neshev D.N. (2017). High-bit rate ultra-compact light routing with mode-selective on-chip nanoantennas. Sci. Adv..

[25] Novotny L., Hecht B. (2006). Principles of Nano-Optics.

[26] Garrido Alzar C.L., Martinez M.A.G., Nussenzveig P. (2002). Classical analog of electromagnetically induced transparency. Am. J. Phys..

[27] Haus H.A. (1983). Waves and Fields in Optoelectronics.

[28] Limonov M.F., Rybin M.V., Poddubny A.N., Kivshar Y.S. (2017). Fano resonances in photonics. Nat. Photon..

[29] Yang Y., Kravchenko I.I., Briggs D.P., Valentine J. (2014). All-dielectric metasurface analogue of electromagnetically induced transparency. Nat. Commun..

[30] Anisimov P.M., Dowling J.P., Sanders B.C. (2011). Objectively discerning Autler-Townes splitting from electromagnetically induced transparency. Phys. Rev. Lett..

[31] Peng B., Şahin Kaya Ö., Chen W., Nori F., Yang L. (2014). What is and what is not electromagnetically induced transparency in whispering-gallery microcavities. Nat. Commun..

[32] Han Z., Bozhevolnyi S.I. (2011). Plasmon-induced transparency with detuned ultracompact Fabry-Perot resonators in integrated plasmonic devices. Opt. Express.

[33] Zhang Z., Yang J., He X., Han Y., Zhang J., Huang J., Chen D., Xu S. (2018). Active enhancement of slow light based on plasmon-induced transparency with gain materials. Materials.

[34] Niu X., Hu X., Yan Q., Zhu J., Cheng H., Huang Y., Lu C., Fu Y., Gong Q. (2019). Plasmon-induced transparency effect for ultracompact on-chip devices. Nanophotonics.

[35] Sun L., Wang J., Wang Y., Liu H., Liu C., Gao S. (2015). Electromagnetically induced transparency of double-groove shaped plasmonic waveguide. Optik.

[36] Shen X., Wang Y., Chen Q., Wu X. (2015). Detuned square ring resonators for multiple plasmon-induced transparencies in metal–insulator–metal waveguide. Appl. Phys. Express.

[37] Xiong C., Li H., Xu H., Zhao M., Zhang B., Liu C., Wu K. (2019). Coupling effects in single-mode and multimode resonator-coupled system. Opt. Express.

[38] Shen X., Wang Y., Yan X., Yuan L., Sang T. (2016). Transmission characteristics and transmission line model of a metal-insulator-metal waveguide with a stub modified by cuts. Appl. Opt..

[39] Ortuño R., Cortijo M., Martínez A. (2017). Fano resonances and electromagnetically induced transparency in silicon waveguides loaded with plasmonic nanoresonators. J. Opt..

[40] Vyas H., Hegde R. (2017). The Fano signature in the optical response of a waveguide-excited compound plasmonic nanoantenna. arXiv.

[41] Tellez-Limon R., Bahari B., Hsu L., Park J.H., Kodigala A., Kanté B. (2016). Integrated metaphotonics: Symmetries and confined excitation of LSP resonances in a single metallic nanoparticle. Opt. Express.

[42] Espinosa-Soria A., Griol A., Martínez A. (2016). Experimental measurement of plasmonic nanostructures embedded in silicon waveguide gaps. Opt. Express.

[43] Downing C.A., Martín-Moreno L. (2021). Polaritonic Tamm states induced by cavity photons. Nanophotonics.

[44] Yan M. (2019). Complex-k modes of plasmonic chain waveguides. J. Phys. Commun..

[45] Vial A., Grimault A.S., Macías D., Barchiesi D., de la Chapelle M.L. (2005). Improved analytical fit of gold dispersion: Application to the modeling of extinction spectra with a finite-difference time-domain method. Phys. Rev. B.

[46] Wittig T., Schuhmann R., Weiland T. (2006). Model order reduction for large systems in computational electromagnetics. Linear Algebra Its Appl..

[47] He X.J., Wang L., Wang J.M., Tian X.H., Jiang J.X., Geng Z.X. (2013). Electromagnetically induced transparency in planar complementary metamaterial for refractive index sensing applications. Appl. Phys..

[48] Marangos J.P. (1998). Electromagnetically induced transparency. J. Mod. Opt..

[49] Fleischhauer M., Imamoglu A., Marangos J.P. (2005). Electromagnetically induced transparency: Optics in coherent media. Rev. Mod. Phys..

